# *N*-(3-Chloro­phen­yl)acetamide

**DOI:** 10.1107/S1600536807068808

**Published:** 2008-01-09

**Authors:** B. Thimme Gowda, Sabine Foro, Hartmut Fuess

**Affiliations:** a Department of Chemistry, Mangalore University, Mangalagangotri 574 199, Mangalore, India; b Institute of Materials Science, Darmstadt University of Technology, Petersenstrasse 23, D-64287 Darmstadt, Germany

## Abstract

The conformation of the N—H bond in the structure of the title compound (3CPA), C_8_H_8_ClNO, is *anti* to the *meta*-chloro substituent, in contrast to the *syn* conformation observed for the *ortho*-chloro substituent in *N*-(2-chloro­phen­yl)acetamide, *syn* to both the *ortho* and *meta* chloro substituents in *N*-(2,3-dichloro­phen­yl)acetamide, and *syn* to the *ortho* chloro substituent in *N*-(2,4-dichloro­phen­yl)acetamide. There are two mol­ecules, linked by an N—H⋯O hydrogen bond, in the asymmetric unit of 3CPA. The bond parameters in 3CPA are similar to those of other acetanilides and the mol­ecules are packed into chains through inter­molecular N—H⋯O hydrogen bonds.

## Related literature

For related literature, see: Gowda *et al.* (2006 ▶); Gowda, Foro & Fuess (2007 ▶); Gowda, Svoboda & Fuess (2007 ▶); Pies *et al.* (1971 ▶).
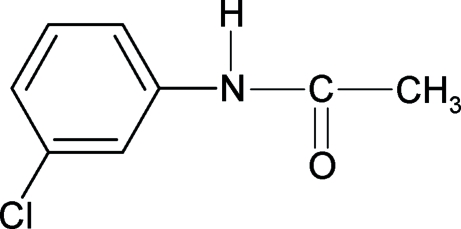


## Experimental

### 

#### Crystal data

C_8_H_8_ClNO
*M* *_r_* = 169.60
Orthorhombic, 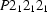

*a* = 4.8468 (8) Å
*b* = 18.562 (2) Å
*c* = 18.852 (3) Å
*V* = 1696.0 (4) Å^3^
*Z* = 8
Cu *K*α radiation
μ = 3.51 mm^−1^
*T* = 299 (2) K
0.60 × 0.15 × 0.08 mm

#### Data collection

Enraf–Nonius CAD-4 diffractometer
Absorption correction: ψ scan (North *et al.*, 1968 ▶) *T*_min_ = 0.225, *T*_max_ = 0.756
3119 measured reflections
2780 independent reflections
2098 reflections with *I* > 2σ(*I*)
*R*_int_ = 0.019
3 standard reflections frequency: 120 min intensity decay: 2.0%

#### Refinement

*R*[*F* ^2^ > 2σ(*F* ^2^)] = 0.082
*wR*(*F* ^2^) = 0.224
*S* = 1.07
2780 reflections
207 parameters
2 restraints
H atoms treated by a mixture of independent and constrained refinement
Δρ_max_ = 0.43 e Å^−3^
Δρ_min_ = −0.59 e Å^−3^
Absolute structure: Flack (1983 ▶), 987 Friedel pairs
Flack parameter: 0.00 (4)

### 

Data collection: *CAD-4-PC* (Enraf–Nonius, 1996 ▶); cell refinement: *CAD-4-PC*; data reduction: *REDU4* (Stoe & Cie, 1987 ▶); program(s) used to solve structure: *SHELXS97* (Sheldrick, 2008 ▶); program(s) used to refine structure: *SHELXL97* (Sheldrick, 2008 ▶); molecular graphics: *ORTEP-3 for Windows* (Farrugia, 1997 ▶) and *PLATON* (Spek, 2003 ▶); software used to prepare material for publication: *SHELXL97*.

## Supplementary Material

Crystal structure: contains datablocks I, global. DOI: 10.1107/S1600536807068808/dn2306sup1.cif

Structure factors: contains datablocks I. DOI: 10.1107/S1600536807068808/dn2306Isup2.hkl

Additional supplementary materials:  crystallographic information; 3D view; checkCIF report

## Figures and Tables

**Table 1:** Hydrogen-bond geometry (Å, °)

*D*—H⋯*A*	*D*—H	H⋯*A*	*D*⋯*A*	*D*—H⋯*A*
N1—H1N⋯O2^i^	0.86 (2)	2.00 (2)	2.846 (5)	166 (6)
N2—H2N⋯O1	0.830 (19)	2.11 (2)	2.927 (5)	167 (6)

Symmetry code: (i) 
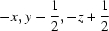
.

**Fig. 1.**
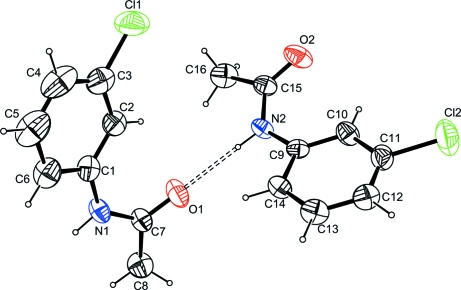
Molecular structure of the title compound, showing the atom labeling scheme. Displacement ellipsoids are drawn at the 30% probability level. Hydrogen bond is shown as dashed line. H atoms are represented as small spheres of arbitrary radii.

**Fig. 2.**
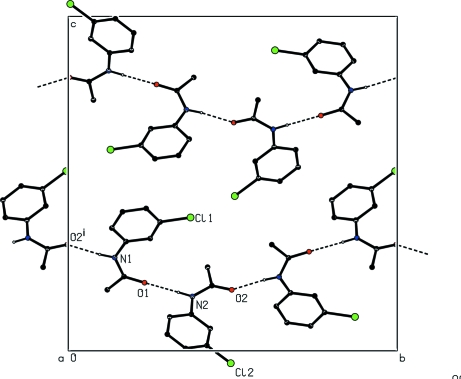
Molecular packing of the title compound with hydrogen bonding shown as dashed lines.H atoms not involved in hydrogen bondings have been omitted for clarity. [Symmetry code: (i) -x, y - 1/2, 1/2 - z]

## supplementary crystallographic information

### Comment

In the present work, the structure of *N*-(3-chlorophenyl)-acetamide (3CPA) has been determined to study the effect of substituents on the structures of *N*-aromatic amides (Gowda, Foro & Fuess, 2007; Gowda, Svoboda & Fuess, 2007). The conformation of the N—H bond in the structure of 3CPA (Fig. 1) is anti to the *meta*-chloro substituent in contrast to the *syn* conformation observed for the *ortho*-chloro substituent in *N*-(2-chlorophenyl)-acetamide (2CPA)(Gowda, Svoboda & Fuess, 2007), *syn* to both the *ortho* and *meta* Chloro substituents in *N*-(2,3-dichlorophenyl)-acetamide(23DCPA)(Gowda, Foro & Fuess, 2007) and *syn* to the *ortho*-chloro substituent in *N*-(2,4-dichlorophenyl)-acetamide (24DCPA)(Gowda, Svoboda & Fuess, 2007). The structure of 3CPA has two molecules linked by N—H···O hydrogen bond in its asymmetric unit. The geometric parameters of 3CPA are similar to those of 2CPA, 23DCPA, 24DCPA and other acetanilides (Gowda, Foro & Fuess, 2007; Gowda, Svoboda & Fuess, 2007). The molecules are linked by hydrogen bonds, N1H1NO2 and N2H2NO1 with respective H1N O2 and H2N O1 lenghts of 2.00 and 2.11 Å, and the angles, N1 H1N O2 and N2 H2N O1 of 166 and 167 °, respectively (Table 1 & Fig. 2).

### Experimental

The title compound was prepared according to the literature method (Gowda *et al.*, 2006). The purity of the compound was checked by determining its melting point. The compound was characterized by recording its infrared,NMR and NQR spectra (Gowda *et al.*, 2006 and Pies *et al.*, 1971). Single crystals of the title compound were obtained from an ethanolic solution and used for X-ray diffraction studies at room temperature.

### Refinement

The NH atoms were located in difference map with N—H = 0.83 (2)–0.86 (2) %A. The other H atoms were positioned with idealized geometry using a riding model with C—H = 0.93–0.96 Å A l l H atoms were refined with isotropic displacement parameters (set to 1.2 times of the *U*_eq_ of the parent atom).

### Crystal data

**Table:**

C_8_H_8_ClNO	*F*_000_ = 704
*M**_r_* = 169.60	*D*_x_ = 1.328 Mg m^−^^3^
Orthorhombic, *P*2_1_2_1_2_1_	Cu *K*α radiation λ = 1.54180 Å
Hall symbol: P 2ac 2ab	Cell parameters from 25 reflections
*a* = 4.8468 (8) Å	θ = 4.8–19.8º
*b* = 18.562 (2) Å	µ = 3.51 mm^−^^1^
*c* = 18.852 (3) Å	*T* = 299 (2) K
*V* = 1696.0 (4) Å^3^	Needle, colourless
*Z* = 8	0.60 × 0.15 × 0.08 mm

### Data collection

**Table:**

Enraf–Nonius CAD4 diffractometer	*R*_int_ = 0.019
Radiation source: fine-focus sealed tube	θ_max_ = 67.0º
Monochromator: graphite	θ_min_ = 3.3º
*T* = 299(2) K	*h* = −5→0
ω/2θ scans	*k* = −22→0
Absorption correction: ψ scan(North *et al.*, 1968)	*l* = −22→14
*T*_min_ = 0.225, *T*_max_ = 0.757	3 standard reflections
3119 measured reflections	every 120 min
2780 independent reflections	intensity decay: 2.0%
2098 reflections with *I* > 2σ(*I*)	

### Refinement

**Table:**

Refinement on *F*^2^	Hydrogen site location: inferred from neighbouring sites
Least-squares matrix: full	H atoms treated by a mixture of independent and constrained refinement
*R*[*F*^2^ > 2σ(*F*^2^)] = 0.082	*w* = 1/[σ^2^(*F*_o_^2^) + (0.1656*P*)^2^] where *P* = (*F*_o_^2^ + 2*F*_c_^2^)/3
*wR*(*F*^2^) = 0.224	(Δ/σ)_max_ = 0.002
*S* = 1.07	Δρ_max_ = 0.43 e Å^−^^3^
2780 reflections	Δρ_min_ = −0.59 e Å^−^^3^
207 parameters	Extinction correction: none
2 restraints	Absolute structure: Flack (1983), 987 Friedel pairs
Primary atom site location: structure-invariant direct methods	Flack parameter: 0.00 (4)
Secondary atom site location: difference Fourier map	

### Special details

**Table:**

Geometry. All e.s.d.'s (except the e.s.d. in the dihedral angle between two l.s. planes) are estimated using the full covariance matrix. The cell e.s.d.'s are taken into account individually in the estimation of e.s.d.'s in distances, angles and torsion angles; correlations between e.s.d.'s in cell parameters are only used when they are defined by crystal symmetry. An approximate (isotropic) treatment of cell e.s.d.'s is used for estimating e.s.d.'s involving l.s. planes.
Refinement. Refinement of *F*^2^ against ALL reflections. The weighted *R*-factor *wR* and goodness of fit *S* are based on *F*^2^, conventional *R*-factors *R* are based on *F*, with *F* set to zero for negative *F*^2^. The threshold expression of *F*^2^ > σ(*F*^2^) is used only for calculating *R*-factors(gt) *etc*. and is not relevant to the choice of reflections for refinement. *R*-factors based on *F*^2^ are statistically about twice as large as those based on *F*, and *R*- factors based on ALL data will be even larger.

### Fractional atomic coordinates and isotropic or equivalent isotropic displacement parameters (Å^2^)

**Table:**

	*x*	*y*	*z*	*U*_iso_*/*U*_eq_	
Cl1	0.3831 (6)	0.37160 (11)	0.39860 (11)	0.1367 (10)	
O1	−0.0630 (10)	0.23187 (18)	0.2036 (2)	0.0880 (12)	
N1	0.0399 (8)	0.14104 (19)	0.2789 (2)	0.0641 (10)	
H1N	0.028 (12)	0.0957 (12)	0.288 (3)	0.077*	
C1	0.2186 (9)	0.1757 (2)	0.3267 (3)	0.0609 (11)	
C2	0.2211 (11)	0.2491 (3)	0.3358 (3)	0.0687 (13)	
H2	0.1068	0.2782	0.3084	0.082*	
C3	0.3931 (14)	0.2792 (4)	0.3853 (3)	0.0854 (17)	
C4	0.5720 (14)	0.2367 (6)	0.4257 (4)	0.108 (3)	
H4	0.6915	0.2575	0.4584	0.130*	
C5	0.5665 (16)	0.1649 (6)	0.4158 (4)	0.116 (3)	
H5	0.6851	0.1359	0.4421	0.139*	
C6	0.3871 (13)	0.1324 (4)	0.3669 (3)	0.0868 (16)	
H6	0.3823	0.0825	0.3619	0.104*	
C7	−0.0907 (10)	0.1695 (2)	0.2219 (3)	0.0608 (11)	
C8	−0.2696 (12)	0.1186 (3)	0.1825 (3)	0.0804 (15)	
H8A	−0.2326	0.0703	0.1980	0.121*	
H8B	−0.2325	0.1225	0.1326	0.121*	
H8C	−0.4597	0.1301	0.1913	0.121*	
Cl2	0.6744 (5)	0.49383 (10)	−0.03716 (11)	0.1181 (8)	
O2	0.0909 (9)	0.49682 (17)	0.1824 (2)	0.0865 (12)	
N2	0.1055 (8)	0.37741 (16)	0.1625 (2)	0.0552 (9)	
H2N	0.085 (12)	0.3348 (13)	0.175 (3)	0.066*	
C9	0.3111 (8)	0.3741 (2)	0.1092 (2)	0.0493 (9)	
C10	0.3766 (11)	0.4316 (2)	0.0668 (3)	0.0635 (12)	
H10	0.2864	0.4756	0.0715	0.076*	
C11	0.5868 (12)	0.4215 (3)	0.0158 (3)	0.0679 (13)	
C12	0.7118 (10)	0.3568 (3)	0.0071 (3)	0.0675 (12)	
H12	0.8437	0.3510	−0.0283	0.081*	
C13	0.6462 (10)	0.3011 (3)	0.0493 (3)	0.0673 (13)	
H13	0.7374	0.2573	0.0441	0.081*	
C14	0.4402 (9)	0.3086 (2)	0.1013 (3)	0.0587 (11)	
H14	0.3920	0.2698	0.1300	0.070*	
C15	0.0080 (9)	0.4367 (2)	0.1948 (3)	0.0593 (11)	
C16	−0.2118 (12)	0.4235 (3)	0.2501 (3)	0.0800 (15)	
H16A	−0.2754	0.3746	0.2467	0.120*	
H16B	−0.3636	0.4558	0.2423	0.120*	
H16C	−0.1362	0.4317	0.2964	0.120*	

### Atomic displacement parameters (Å^2^)

**Table:**

	*U*^11^	*U*^22^	*U*^33^	*U*^12^	*U*^13^	*U*^23^
Cl1	0.172 (2)	0.1231 (14)	0.1148 (14)	−0.0737 (15)	0.0260 (16)	−0.0395 (11)
O1	0.094 (3)	0.0659 (19)	0.104 (3)	−0.0177 (19)	−0.023 (2)	0.0274 (19)
N1	0.057 (2)	0.0524 (18)	0.082 (3)	−0.0095 (16)	−0.003 (2)	0.0140 (19)
C1	0.041 (2)	0.075 (3)	0.067 (3)	−0.0049 (19)	0.003 (2)	0.008 (2)
C2	0.058 (3)	0.078 (3)	0.070 (3)	−0.014 (2)	0.005 (3)	0.003 (2)
C3	0.068 (4)	0.115 (4)	0.073 (3)	−0.034 (3)	0.023 (3)	−0.011 (3)
C4	0.053 (3)	0.188 (8)	0.083 (4)	−0.032 (4)	−0.001 (3)	−0.026 (5)
C5	0.075 (4)	0.176 (8)	0.097 (5)	0.034 (5)	−0.025 (4)	−0.007 (5)
C6	0.070 (3)	0.104 (4)	0.086 (4)	0.020 (3)	−0.010 (3)	0.007 (3)
C7	0.054 (2)	0.055 (2)	0.073 (3)	−0.0023 (19)	−0.003 (2)	0.009 (2)
C8	0.062 (3)	0.085 (3)	0.093 (4)	−0.013 (3)	−0.010 (3)	0.001 (3)
Cl2	0.1326 (17)	0.1002 (11)	0.1217 (13)	−0.0127 (11)	0.0357 (13)	0.0404 (10)
O2	0.086 (3)	0.0506 (16)	0.123 (3)	0.0048 (19)	0.018 (2)	−0.0152 (18)
N2	0.0491 (18)	0.0411 (15)	0.075 (2)	−0.0052 (15)	0.0041 (19)	−0.0026 (16)
C9	0.0391 (18)	0.0504 (19)	0.058 (2)	−0.0029 (15)	−0.0012 (19)	−0.0094 (18)
C10	0.058 (3)	0.051 (2)	0.081 (3)	0.005 (2)	0.004 (2)	0.006 (2)
C11	0.067 (3)	0.071 (3)	0.066 (3)	−0.016 (2)	0.001 (3)	0.007 (2)
C12	0.047 (2)	0.081 (3)	0.075 (3)	−0.002 (2)	0.007 (2)	−0.007 (3)
C13	0.040 (2)	0.072 (3)	0.090 (3)	0.009 (2)	0.008 (2)	−0.009 (2)
C14	0.051 (2)	0.050 (2)	0.075 (3)	−0.0008 (18)	−0.003 (2)	0.001 (2)
C15	0.052 (2)	0.053 (2)	0.073 (3)	0.0010 (18)	0.000 (2)	−0.007 (2)
C16	0.054 (3)	0.089 (3)	0.097 (4)	0.004 (2)	0.012 (3)	−0.015 (3)

### Geometric parameters (Å, °)

**Table:**

Cl1—C3	1.735 (7)	Cl2—C11	1.726 (5)
O1—C7	1.215 (5)	O2—C15	1.209 (6)
N1—C7	1.355 (6)	N2—C15	1.343 (5)
N1—C1	1.406 (6)	N2—C9	1.416 (5)
N1—H1N	0.86 (2)	N2—H2N	0.830 (19)
C1—C2	1.373 (7)	C9—C10	1.371 (6)
C1—C6	1.375 (7)	C9—C14	1.376 (6)
C2—C3	1.371 (8)	C10—C11	1.414 (8)
C2—H2	0.9300	C10—H10	0.9300
C3—C4	1.397 (11)	C11—C12	1.354 (7)
C4—C5	1.347 (12)	C12—C13	1.343 (7)
C4—H4	0.9300	C12—H12	0.9300
C5—C6	1.403 (10)	C13—C14	1.407 (7)
C5—H5	0.9300	C13—H13	0.9300
C6—H6	0.9300	C14—H14	0.9300
C7—C8	1.482 (7)	C15—C16	1.511 (7)
C8—H8A	0.9600	C16—H16A	0.9600
C8—H8B	0.9600	C16—H16B	0.9600
C8—H8C	0.9600	C16—H16C	0.9600
			
C7—N1—C1	128.1 (4)	C15—N2—C9	127.2 (4)
C7—N1—H1N	121 (4)	C15—N2—H2N	128 (4)
C1—N1—H1N	111 (4)	C9—N2—H2N	104 (4)
C2—C1—C6	120.5 (5)	C10—C9—C14	121.3 (4)
C2—C1—N1	122.6 (4)	C10—C9—N2	122.9 (4)
C6—C1—N1	116.8 (5)	C14—C9—N2	115.8 (4)
C3—C2—C1	119.6 (6)	C9—C10—C11	117.4 (4)
C3—C2—H2	120.2	C9—C10—H10	121.3
C1—C2—H2	120.2	C11—C10—H10	121.3
C2—C3—C4	121.3 (7)	C12—C11—C10	121.5 (4)
C2—C3—Cl1	118.9 (6)	C12—C11—Cl2	120.6 (4)
C4—C3—Cl1	119.8 (6)	C10—C11—Cl2	117.8 (4)
C5—C4—C3	118.1 (6)	C13—C12—C11	120.3 (5)
C5—C4—H4	121.0	C13—C12—H12	119.8
C3—C4—H4	121.0	C11—C12—H12	119.8
C4—C5—C6	121.9 (7)	C12—C13—C14	120.4 (4)
C4—C5—H5	119.0	C12—C13—H13	119.8
C6—C5—H5	119.0	C14—C13—H13	119.8
C1—C6—C5	118.6 (6)	C9—C14—C13	119.0 (4)
C1—C6—H6	120.7	C9—C14—H14	120.5
C5—C6—H6	120.7	C13—C14—H14	120.5
O1—C7—N1	123.0 (5)	O2—C15—N2	123.5 (5)
O1—C7—C8	122.0 (5)	O2—C15—C16	121.1 (4)
N1—C7—C8	114.9 (4)	N2—C15—C16	115.4 (4)
C7—C8—H8A	109.5	C15—C16—H16A	109.5
C7—C8—H8B	109.5	C15—C16—H16B	109.5
H8A—C8—H8B	109.5	H16A—C16—H16B	109.5
C7—C8—H8C	109.5	C15—C16—H16C	109.5
H8A—C8—H8C	109.5	H16A—C16—H16C	109.5
H8B—C8—H8C	109.5	H16B—C16—H16C	109.5
			
C7—N1—C1—C2	21.1 (8)	C15—N2—C9—C10	−22.1 (7)
C7—N1—C1—C6	−161.3 (5)	C15—N2—C9—C14	159.1 (5)
C6—C1—C2—C3	0.0 (8)	C14—C9—C10—C11	−1.5 (7)
N1—C1—C2—C3	177.5 (5)	N2—C9—C10—C11	179.8 (4)
C1—C2—C3—C4	1.8 (8)	C9—C10—C11—C12	2.4 (8)
C1—C2—C3—Cl1	−177.4 (4)	C9—C10—C11—Cl2	−179.1 (4)
C2—C3—C4—C5	−1.6 (10)	C10—C11—C12—C13	−2.8 (8)
Cl1—C3—C4—C5	177.5 (6)	Cl2—C11—C12—C13	178.8 (4)
C3—C4—C5—C6	−0.3 (12)	C11—C12—C13—C14	2.2 (8)
C2—C1—C6—C5	−1.8 (9)	C10—C9—C14—C13	0.9 (7)
N1—C1—C6—C5	−179.4 (6)	N2—C9—C14—C13	179.8 (4)
C4—C5—C6—C1	2.0 (11)	C12—C13—C14—C9	−1.3 (7)
C1—N1—C7—O1	1.5 (8)	C9—N2—C15—O2	−1.2 (8)
C1—N1—C7—C8	−179.1 (5)	C9—N2—C15—C16	−179.8 (4)

### Hydrogen-bond geometry (Å, °)

**Table:**

*D*—H···*A*	*D*—H	H···*A*	*D*···*A*	*D*—H···*A*
N1—H1N···O2^i^	0.86 (2)	2.00 (2)	2.846 (5)	166 (6)
N2—H2N···O1	0.830 (19)	2.11 (2)	2.927 (5)	167 (6)

Symmetry codes: (i) −*x*, *y*−1/2, −*z*+1/2.
